# Co-culturing human prostate carcinoma cells with hepatocytes leads to increased expression of E-cadherin

**DOI:** 10.1038/sj.bjc.6603700

**Published:** 2007-04-03

**Authors:** C C Yates, C R Shepard, D B Stolz, A Wells

**Affiliations:** 1 Department of Pathology, University of Pittsburgh, and Pittsburgh VAMC, Pittsburgh, PA 15213, USA; 2 Department of Cell Biology, University of Pittsburgh, and Pittsburgh VAMC, Pittsburgh, PA 15213, USA

**Keywords:** EGF receptor, centrifugal assay for cell adhesion, heterotypic cell–cell adhesion, epithelial–mesenchymal transition

## Abstract

Metastasis is a multi-step process wherein tumour cells detach from the primary mass, migrate through barrier matrices, gain access to conduits to disseminate, and subsequently survive and proliferate in an ectopic site. During the initial invasion stage, prostate carcinoma cells undergo epithelial–mesenchymal-like transition with gain of autocrine signalling and loss of E-cadherin, hallmarks that appear to enable invasion and dissemination. However, some metastases express E-cadherin, and we found close connections between prostate carcinoma cells and hepatocytes in a liver microtissue bioreactor. We hypothesise that phenotypic plasticity occurs late in prostate cancer progression at the site of ectopic seeding. Immunofluorescence staining for E-cadherin in co-cultures of hepatocytes and DU-145 prostate cancer cells revealed E-cadherin upregulation at peripheral sites of contact by day 2 of co-culture; E-cadherin expression also increased in PC-3 cells in co-culture. These carcinoma cells bound to hepatocytes in an E-cadherin-dependent manner. Although the signals by which the hepatocytes elicited E-cadherin expression remain undetermined, it appeared related to downregulation of epidermal growth factor receptor (EGFR) signalling. Inhibition of autocrine EGFR signalling increased E-cadherin expression and cell–cell heterotypic adhesion; further, expression of a downregulation-resistant EGFR variant prevented E-cadherin upregulation. These findings were supported by finding E-cadherin and catenins but not activated EGFR in human prostate metastases to the liver. We conclude that the term epithelial–mesenchymal transition only summarises the transient downregulation of E-cadherin for invasion with re-expression of E-cadherin being a physiological consequence of metastatic seeding.

Metastasis causes the vast majority of the morbidity and mortality from cancer. However, our understanding of the multi-step cascade of events that must occur for successful attachment and subsequent metastasis has not been completely elucidated, as few systems are available to examine the intermediary dynamic steps that occur during progression (Condeelis and Segall, 2003; Yates *et al*, 2007). Only now are investigators defining the changes needed for both a cell to escape from the primary tumour and subsequently to allow for ectopic survival and proliferation.

An essential step for cells to migrate from the primary tumour mass is the loss of epithelial cell–cell adhesions and subsequent acquisition of a mesenchymal-like migratory and invasive phenotype, generally referred to as epithelial–mesenchymal transition (EMT) (Thiery, 2002; Ackland *et al*, 2003; Bates and Mercurio, 2005). Central to this dissociation from the primary tumour is altered or loss of E-cadherin expression (Davies *et al*, 2000; Lowy *et al*, 2002) a key attribute that may define the EMT. E-cadherin is a calcium (Ca^2+^)-dependent transmembrane cell surface glycoprotein. E-cadherin rapidly localises on the cell surface to regions of contact, usually resulting in homotypic cell–cell binding; this fosters the maintenance of normal cellular structure. Recently, reports have shown that aberrant loss or downregulation of E-cadherin in carcinomas may result from reversible epigenetic events (Graff *et al*, 1995; Lind *et al*, 2004) or growth factor-mediated downregulation (Hurtubise and Momparler, 2004; Wheeler, 2005), suggesting that loss of this tumour suppressor may be reversible. That this reversal might happen has been suggested by the findings that secondary tumour metastases express E-cadherin (Brabletz *et al*, 2001; Rubin *et al*, 2001). However, that this expression at the distal site represents re-expression of E-cadherin has not been demonstrated.

Activation of the epidermal growth factor receptor (EGFR) downregulates E-cadherin expression (Jawhari *et al*, 1999; Ackland *et al*, 2003). This suggested that autocrine EGFR signalling present in prostate carcinomas may contribute to E-cadherin repression in these tumours. This was supported by our recent finding that pharmacological abrogation of EGFR signalling in prostate carcinoma cells reverses decreased E-cadherin expression rendering these cells less invasive and more cohesive (Jawhari *et al*, 1999; Yates *et al*, 2005). This re-expression of E-cadherin is similarly observed by close contacts of DU-145 prostate carcinoma cells when cultured within the liver microenvironment (Yates *et al*, 2007). As prostate cancer metastasises to the liver in over half of all patients with metastatic disease (Ewing, 1922; Shah *et al*, 2004), we sought to explore the correlative expression levels of EGFR and E-cadherin in the presence of parenchymal cells of the liver. We show, herein, that hepatocytes elicit E-cadherin expression in prostate carcinoma cells concomitant with downregulation of EGFR signalling.

## MATERIALS AND METHODS

### Materials

The primary antibodies used were mouse monoclonal antibodies selective for human E-cadherin, human cytokeratin 18 and human-specific controls tubulin (Santa Cruz Biotechnology, Santa Cruz, CA, USA). Primary antibodies to phospho-EGFR (Cell Signaling, Danvers, MA, USA), *α*-catenin, *β*-catenin, p120 (BD Biosciences, San Jose, CA, USA) and vimentin (Dako Laboratories, Carpinteria, CA, USA). Polyclonal antibody to EGFR was used for immunohistochemistry (Santa Cruz). Secondary antibodies for immunofluorescence dyes or horseradish peroxidase-conjugated were obtained from (Jackson Laboratories, Bar Harbor, ME, USA). Anti-E-cadherin blocking antibody (SHE78-7) was obtained from Zymed Laboratories (South San Francisco, CA, USA). The EGFR inhibitor PD153035 and primary antibody to human EGFR (Ab-1), were obtained from Calbiochem (San Diego, CA, USA). Other reagents were obtained from Sigma (St Louis, MO, USA).

### Cell lines

The androgen-independent DU-145 and PC-3 human prostate carcinoma cell line were originally derived from a brain and bone metastasis of a human prostate adenocarcinoma, respectively. A transmodulation-resistant EGFR construct was generated by replacing the PKC-target threonine at amino acid 654 with alanine (A654) and transfected into DU-145 cells (Yates *et al*, 2005). Prostate cancer cells lines were cultured as described previously (Yates *et al*, 2005). Red fluorescent protein (RFP) was stably transfected with ds-Red (Clontech, Mountain View, CA, USA) vector containing a neomycin resistance gene in DU-145 cells. RFP expressing cells were selected and maintained in DMEM with 10% FBS and in the presence of 1000 mg ml^−1^ G418 until used for experimentation.

### Reverse transcription-PCR

Total RNA was extracted using TRIzol (Invitrogen, Carlsbad, CA, USA). RT reaction was performed using total RNA as a template and an RT for PCR kit (Invitrogen). PCR amplification was carried out with the following primers: human-specific E-cadherin primers, 5′-gacacccgattcaaagtggg-3′ and 5′-gtctctcttctgtcttctgag-3′; showed no homology to rat E-cadherin and 30.2% homology to mouse E-cadherin. GAPDH primer kit 5′-ccacccatggcaaattccatggca-3′ and 5′-tctaacggcaggtcaggtccacc-3′ (Stratagene, Cedar Creek, TX, USA) recognises both human and rodent sequences.

### Hepatocytes

Primary rat hepatocytes, >90% purified, were obtained by collagenase perfusion (Seglen, 1976) of 150–230 g male eGFP (enhanced green fluorescent protein)-transgenic and WT (wild-type) Sprague–Dawley rats, originally generated by Dr Masaru Okabe (Genome Information Research Center, University of Osaka, Osaka, Japan) and were generously provided by Japan SLC, Inc. (Hamamatsu, Japan). Use of rat hepatocytes enables the identification of the human prostate carcinoma cell proteins using human-specific antibodies. The cells were collected and maintained in Hepatocyte Growth Media (HGM) medium before and during co-culture (Yates *et al*, 2007). These studies were approved by the University of Pittsburgh IACUC committee.

### Co-cultures

Initial co-cultures consisted of 50 000 cells cm^−2^ of freshly isolated hepatocytes and 2000 cells cm^−2^ prostate cancer cells. Co-cultures were maintained in serum-free HGM (Yates *et al*, 2007), and plated on tissue culture dishes pre-treated with 10% collagen (Upstate, Charlottesville, VA, USA).

### Centrifugal assay for cell adhesion

This assay is a modification of the McClay and Giacolmello assays (McClay *et al*, 1981). Cancer cells were non-enzymatically dissociated and labelled with 5 *μ*M Calcein AM (Molecular Probes, Carlsbad, CA, USA). Labelled cancer cells were seeded at a density of 42 000 cells well^−1^ in 96-well plates containing a densely confluent hepatocyte monolayer. The plates were centrifuged for <60s at 50 **g** to pellet the cancer cells onto the hepatic monolayer, then incubated at 37°C. At defined times, the plates were inverted and centrifuged at 600 **g** for 5 min and then gently washed to remove unbound cells from the hepatocyte monolayer. Fluorescence was measured with a 494/517 bandpass filter set-up from the bottom of the plate by a TECAN Spectra-Fluor plate fluorometer. Absolute emission measurements were background subtracted.

### Tissue specimens

Formalin-fixed paraffin-embedded tissues were obtained from the University of Pittsburgh Tumor Bank. We found only two well-defined prostate adenocarcinomas with liver metastases and none from the Cooperative Human Tumor Network, irrespective of other criteria. These studies, blinded for all personal identifiers, were categorised as exemption 4e by the University of Pittsburgh IRB.

### Statistical analysis

Statistics for all experiments were performed using the Sigma Plot statistical program (Jandel Scientific, Chicago, IL, USA). Independent Student's *t*-test was utilised to determine a statistical difference between experimental and the controls for individual experiments, with significance generally informed at *P*<0.05.

## RESULTS

E-cadherin is generally lost from the primary tumour cells of metastatic carcinomas, with the result that these cells can now detach from the primary tumour mass and attain a nonpolarised, migratory phenotype (Bates and Mercurio, 2005). Unlike many other tumour suppressors, the E-cadherin gene is neither deleted nor mutated, but rather the downregulation appears to result from epigenetic signals, opening the possibility that this loss of expression may be reverted later during the metastatic cascade (Yates *et al*, 2007). Intriguingly, E-cadherin expression has been noted on metastases of human carcinomas (Rubin *et al*, 2001). Thus, we proposed that E-cadherin re-expression coincided with distant metastases, enabling the prostate carcinoma cells to interact with the ectopic parenchyma (Yates *et al*, 2007). We tested this postulate using liver hepatocytes, as liver is the epithelial organ most commonly colonised by metastatic prostate carcinoma (Ewing, 1922; Shah *et al*, 2004).

**Figure 1:**
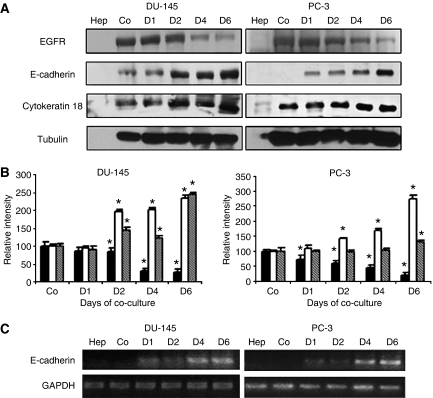
Co-culture of human prostate cancer cell with rat hepatocytes reversed E-cadherin expression. DU-145 or PC-3 (**A**) cells were co-cultured in the presence of primary rat hepatocytes over a 6-day period. Hepatocytes and single cultures were lysed before co-cultures. On days 1, 2, 4 and 6, co-culture lysates were immunoblotted with indicated human selective antibodies: anti-E-cadherin, anti-EGFR antibody, anti-cytokeratin 18 and anti-tubulin (as the loading control). (**B**) Densitometry of immunoblots in DU-145 and PC-3 cells co-cultures (▪) EGFR, (□) E-cadherin () and Cytokeratin 18; shown are the mean±s.d. of three blots with day 0 being at 100 (^*^P<0.05 from C0). Cellular levels of E-cadherin mRNA in DU-145 or PC-3 (**C**) cells were analysed by RT-PCR using GAPDH as a loading control. In **A** and **B**, Co (for control) are an equal number of prostate carcinoma cells incubated for 1 day in the absence of hepatocytes. The Hep (hepatocytes) were an equal number of hepatocytes as for the co-cultures. The first three lanes were all lysed at the same time. Shown are representative of at least three experiments.

We found that co-culturing DU-145 and PC-3 carcinoma cells with rat hepatocytes resulted in increased E-cadherin expression and decreased EGFR expression (Figure 1A). This occurred over a 6-day period in both cell types, with significant differences by day 2 (Figure 1A and B). The increase in E-cadherin protein levels was reflected by similarly elevated mRNA levels (Figure 1B), though the appearance of mRNA may lag by about 1 day. This time differential may imply two levels of control – one at protein degradation and the other at *de novo* transcriptional potential.

Since loss of E-cadherin and increased EGFR signalling are known markers of invasive mesenchymal cancer cells (Wong and Gumbiner, 2003), we asked if the E-cadherin re-expression and EGFR decreases were accompanied with another known epithelial cell marker. Cytokeratin 18 expression, a marker of mature epithelial cells, increased over the 6-day period in both DU-145 and PC-3 cell lines suggesting a reversion of mesenchymal phenotype characteristic of these cell lines (Figure 1A). This implied a generalised reversion to a more differentiated phenotype in the presence of hepatocytes.

**Figure 2:**
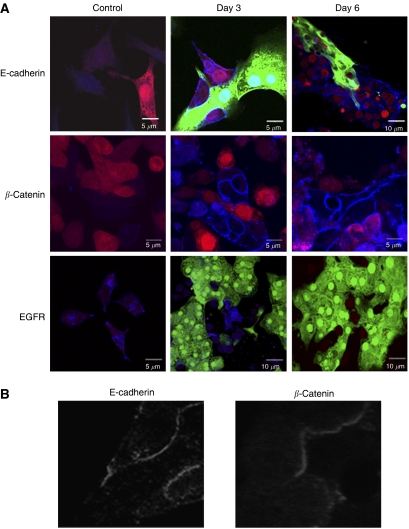
Immunofluorescence of co-cultures show subcellular location of E-cadherin re-expression. (**A**) Immunofluorescence of DU-145 RFP (red) and GFP (green) primary rat hepatocytes were stained with human-specific anti-E-cadherin, pan-species anti-*β*-catenin or human-specific anti-EGFR antibody. Cy5 secondary antibody (blue) was used for respective primary antibodies. Note the gain of blue (E-cadherin) in the RFP/red prostate cells in the top and middle rows, and the loss of blue (EGFR) in the bottom row. (**B**) The blue channel only of the lower left inset in the E-cadherin and *β*-catenin staining on day 2 is shown in black and white to demonstrate the localisation of the human E-cadherin in the prostate carcinoma cells to the interface with the hepatocytes. In the middle row, the hepatocytes were from WT and not GFP rats, so as not to interfere with the antibody staining as the anti-*β*-catenin detected both human and rat. However, the presence of *β*-catenin upregulation in the DU-145 cells is noted by a violet color and the membranous pattern at the hepatocyte–prostate carcinoma cell interface. Shown are representative of at least three experiments.

**Figure 3:**
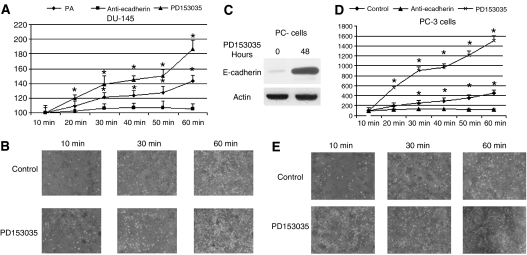
Human prostate cancer cells form E-cadherin-mediated heterotypic interactions with hepatocytes. DU-145 (**A** and **B**) cells or PC-3 (**C**–**E**) cells fluorescently labelled with Calcein A were incubated for 48 h with PD153035 or E-cadherin blocking antibody and seeded onto a monolayer of hepatocytes from non-GFP-expressing rats to analyse their ability to adhere. Cell binding was assessed by fluorescent intensity using a plate fluorometer and visually verified under a fluorescent light microscope. Y-axis is arbitrary fluorescent units. Data represent mean of three experiments performed in triplicate; s.e. ^*^P<0.05. Shown are sample representative fields to show the bound tumour cells (converted to white dots) overlying the hepatocytes in **B** and **E**. (**C**) PC-3 cells were exposed to PD153035 for 48 h with a resultant upregulation of E-cadherin as shown by immunoblotting. This is similar to our previously published finding with DU145 cells (Yates *et al*, 2005).

Looking more closely at the subcellular localisation of this E-cadherin and EGFR expression, freshly isolated GFP-expressing primary rat hepatocytes were allowed to adhere 24 h before seeding of the RFP-expressing prostate cancer cells. As expected, immunofluorescence showed increases in E-cadherin and *β*-catenin and inversely related decreases in EGFR expression in the presence of hepatocytes. Unexpectedly however, early co-cultures revealed E-cadherin expression at the prostate cancer cell periphery juxtaposed to the hepatocytes during attachment (Figure 2A and B). *β*-Catenin was also localised to the membrane area in these cells further suggesting a functional E-cadherin linkage at the interface of these two cell types. To our knowledge, E-cadherin interaction among different cell types has not been noted previously, thus we consider these interactions as potentially heterotypic in terms of cell types. While various other proteins (e.g. selectins and integrins) have been implicated in carcinoma attachment, heterotypic cell–cell interaction via E-cadherin has not. Therefore, the ability of prostate cancer cells to utilise E-cadherin to bind to hepatocytes was tested by assessing cell–cell adhesion between adherent hepatocytes and prostate carcinoma cells using the centrifugal assay for cell adhesion (McClay *et al*, 1981). Both DU-145 and PC-3 cells, calcein-AM-labelled, bound to the hepatocytes to a limited but statistically significant degree, and this binding was eliminated by an E-cadherin blocking antibody (Figure 3A–E). As the cell–cell adhesion of the prostate carcinoma cells was limited, though real, we sought to increase the levels of E-cadherin on these cells. For this we used the EGFR kinase inhibitor, PD153035, that we had shown earlier to promote E-cadherin expression in DU-145 cells within 24–48 h (Yates *et al*, 2005), and confirmed in PC-3 cells at 48 h (Figure 3C). This 48-h pretreatment with PD153035 increased prostate carcinoma cell binding to the hepatocyte monolayer (Figure 3A–E).

**Figure 4:**
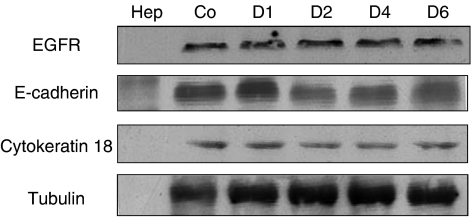
DU-145 cells expressing a PKC transattenuation-resistant EGFR (A654) are resistant to hepatocyte-induced E-cadherin re-expression. DU-145 A654 cells co-culture lysates were immunoblotted with an antibody selective for human E-cadherin, EGFR, cytokeratin 18 or tubulin. The legend is as with Figure 1, Co (control) DU145 A654 cells and Hep (hepatocytes) only. Shown is one of two representative blot series.

The above findings implicate EGFR signalling as potentially negatively regulating E-cadherin levels. As this was reminiscent of the situation with LHRH receptor transmodulation of autocrine EGFR signalling in DU-145 cells (Yates *et al*, 2005), we asked whether a similar situation might be functioning in this setting. We expressed the PKC transmodulation-resistant EGFR A654 variant (Welsh *et al*, 1991) in the DU-145 cells. Co-culturing these cells with hepatocytes did not lead to decreases in EGFR or increases in E-cadherin and cytokeratin 18 expression (Figure 4). If anything, the DU-145 A654 cells exhibited a decrease in E-cadherin expression over 6-day period, which would be expected since EGFR signalling continued unabated, and EGFR activity is well documented as leading to E-cadherin downregulation.

**Figure 5:**
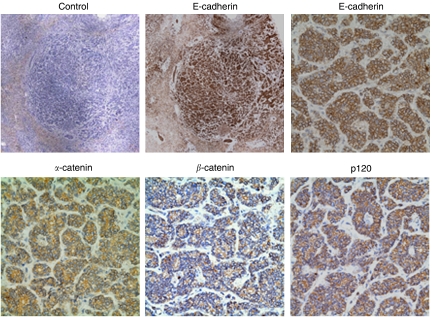
Human prostate cancer metastases to liver show expression of cell–cell adhesion molecules. Formalin-fixed, paraffin-embedded tissues were obtained from two well-defined prostate adenocarcinomas with liver metastasis. Tissues were stained with indicated antibodies, Secondary antibody, anti-mouse only as the staining control (top left; 3700 *μ*m^2^), anti-E-cadherin (top centre; 3700 *μ*m^2^ and top right; 300 *μ*m^2^), anti-*α*-catenin (bottom right; 300 *μ*m^2^), anti-*β*-catenin (bottom centre; 300 *μ*m^2^) and anti-p120 (bottom right; 300 *μ*m^2^). Shown are representative of repeated stainings; the other metastasis presented similar findings.

**Figure 6:**
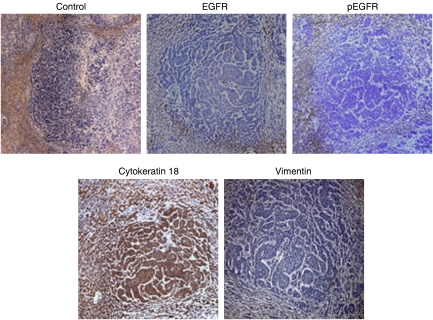
Human prostate cancer metastases show reversion of metastatic markers. Tissues were stained with anti-rabbit (top left; 1400 *μ*m^2^) anti-EGFR (top centre; 1400 *μ*m^2^), anti-phosphotyrosyl-EGFR (activated EGFR) (top right; 1400 *μ*m^2^), anti-vimentin (bottom left; 1400 *μ*m^2^) and anti-cytokeratin 18 (bottom left; 1400 *μ*m^2^). Shown are representative of repeated stainings; the other metastasis presented similar findings.

These data present a potential metastatic target organ-specific regulation of prostate carcinoma cell phenotype. However, to validate whether this may occur in *de novo* human tumours, we obtained human liver tissue from two patients with prostate cancer metastases to the liver and examined the expression of E-cadherin in these tumours by immunohistochemistry. E-cadherin staining was significant in the tumour nodules within the liver (Figure 5A). This increased expression was accompanied by increases in E-cadherin-associated adhesion molecules *α*, *β* and p120 catenin as well (Figure 5A). Central to our model of inverse relationship between E-cadherin expression and EGFR (Yates *et al*, 2005), we were able to observe a lack of total and active EGFR expression in these tumours (Figure 6A). To molecularly determine if these tumours reverted to a more epithelial phenotype, we stained for the epithelial marker cytokeratin 18 and the mesenchymal marker vimentin. Akin to our *in vitro* findings, these tumours largely express cytokeratin and lack vimentin expression (Figure 6B). In the absence of the primary tumours, from which these metastases derived, we cannot state that this represents a reversion in the phenotypic profile, but given the widespread finding of EMT in invasive and metastatic primary tumour, it does suggest that there may be cancer cell phenotypic variability as result of the metastatic microenvironment.

## DISCUSSION

Loss of E-cadherin is so widely observed in advanced carcinomas that the E-cadherin molecule is considered a tumour suppressor (Wong and Gumbiner, 2003). This conception of E-cadherin is supported by forced re-expression of E-cadherin resulting in diminished tumourigenic potential in experimental tumour systems (Jawhari *et al*, 1999; Yates *et al*, 2005). However, unlike classical tumour suppressors (e.g. p53 and Rb), E-cadherin loss occurs by epigenetic downregulation or transcriptional silencing, rather than genetic deletion or mutation (Graff *et al*, 1995). As the mechanisms which diminish E-cadherin are reversible, this opens the question as to whether there are situations in which re-expression of E-cadherin and subsequent cell–cell adhesion may promote tumour progression. Herein, we provide proof of principle that parenchymal cells from a tissue frequently targeted for prostate carcinoma metastasis, the liver (Ewing, 1922; Lind *et al*, 2004; Shah *et al*, 2004), signal this re-expression of E-cadherin.

Downregulation of E-cadherin occurs frequently in the primary prostate tumour coincident with aggression and spread (Gingrich *et al*, 1996; Kallakury *et al*, 2001). However, expression in distant metastases has been noted in some prostate cancer metastases (Rubin *et al*, 2001) but not in other studies in metastases to the bone (Bryden *et al*, 2002). Our findings herein, appear consistent with the re-expression, though the liver environment is likely substantially different from that of the bone in terms of signals to the hepatocytes and requirements by the metastatic tumour cells. It must also be noted that there is precedence for E-cadherin expression on disseminated carcinomas, as ovarian carcinoma dissemination throughout the peritoneal cavity shows increased levels of E-cadherin and its attendant catenins (Imai *et al*, 2004). Again, though, the applicability to metastatic spread of an organ-confined prostate cancer to a distant-defined organ compartment may not be comparable to the spread of ovarian carcinoma throughout an adjacent open cavity.

The findings herein raise two questions that need to be explored in depth in future investigations. First and most obvious, the mechanism by which the hepatocytes signal for E-cadherin re-expression needs to be defined. Our initial findings strongly suggest a link to downregulation of autocrine EGFR signalling. EGFR levels change inversely to the E-cadherin levels, and direct inhibition of EGFR signalling leads to increased E-cadherin expression (Yates *et al*, 2005). More directly, expression of an EGFR variant that is resistant to PKC-mediated attenuation renders the DU-145 cells impervious to hepatocyte-induced E-cadherin expression. This presages the ultimate issues of the nature of the signals from the hepatocytes and which carcinoma intracellular processes actuate the E-cadherin re-emergence. The intracellular events may be complex and involve both loss of downregulation at the protein level and reversion of transcriptional suppression. That EGFR activity is known to disrupt the E-cadherin/catenin complex leading to E-cadherin protein destabilisation and degradation (Ackland *et al*, 2003) and the impression that E-cadherin protein expression may precede increased mRNA suggest that protein downregulation is part of the answer. Although the intracellular mechanisms of E-cadherin re-expression are undoubtedly complex, these findings are novel and provide insight of prostate cancer cell behavior within a target soft organ.

One caveat that deserves specific mention is that EGFR signalling contributes to numerous aspects of prostate tumour progression. For instance, autocrine EGFR-mediated motility promotes tumour invasion and metastasis and tumour cell proliferation and survival (Wells, 2000). Thus, targeting EGFR kinase may have effects other than promoting the metastatic engraftment proposed herein by the upregulation of E-cadherin. For instance, the recent report of EGFR kinase inhibitors limiting prostate tumour dissemination in experimental models (Angelucci *et al*, 2006) is likely due to inhibiting pathways and cell behaviors before the proposed upregulation of E-cadherin at the metastatic site, though re-expression of E-cadherin at the primary tumour site would also limit spread by preventing initial detachment (Jawhari *et al*, 1999; Lowy *et al*, 2002; Wong and Gumbiner, 2003).

The second question of what selective advantage is conferred by E-cadherin re-expression remains in the speculative realm. Our finding and those reported in the literature (Rubin *et al*, 2001; Kowalski *et al*, 2003) or carcinoma metastases presenting E-cadherin and differentiation-related cytokeratins, along with low to absent levels of vimentin and EGFR (total and active), cannot discern whether these metastases arose from a subset of primary tumour cells that never underwent a phenotypic shift or from the majority of aggressive cells that underwent the so-called epithelial–mesenchymal transition. However, the fact that experimental prostate cancer cell lines, DU-145 and PC-3, exhibited similar phenotypic shifts when cultured with hepatocytes implies a survival or growth advantage in these ectopic sites that is at odds with that seen for the EMT tumour cells in the primary locale. Recently, this has been supported experimentally by the finding that epithelial variants of a bladder carcinoma cell line presented greater seeding of metastatic sites when introduced into the circulation than the mesenchymal parental line, whereas the parental mesenchymal tumour line possessed increased metastatic capabilities from an orthotopic location (Chaffer *et al*, 2006). One can speculate that in a metastatic tumour microenvironment lacking many of the prostate-specific survival signals, E-cadherin-mediated linkages provide critical survival signals, by linking to both other carcinoma cells and even the hepatocytes themselves. Such studies, which lie beyond the realm of the current communication, are underway.

In conclusion, we propose a model in which the differentiation phenotype of metastatic prostate carcinoma cells is plastic in response to the microenvironment. In the primary tumour setting, E-cadherin expression is downregulated as part of the EMT that allows disaggregation from the primary mass enabling invasive migration and distant dissemination. However, once the tumour cells reach the ectopic microenvironment, numerous signals critical for survival and/or growth are absent. The metastatically competent subset of tumour cells interpret initiating signals from the target organ, in this case the liver, to re-express a more differentiated phenotype, or to undergo a mesenchymal–epithelial *reverse* transition. This redifferentiation could provide for both homotypic and heterotypic cell–cell adhesion with concomitant survival signals. While such a redifferentiation would be expected to limit tumour cell proliferation and local invasiveness, it may be key to prevent tumour cell apoptosis in the absence of a supportive orthotopic microenvironment. In-depth exploration is necessary not only to determine if such metastatic carcinoma redifferentiation occurs in *de novo* metastasis of human tumours, but also to explain the relative resistance to chemotherapy of metastases and even the concept of tumour dormancy.

## References

[bib1] Ackland ML, Newgreen DF, Fridman M, Waltham MC, Arvanitis A, Minichiello J, Price JT, Thompson EW (2003) Epidermal growth factor-induced epithelio–mesenchymal transition in human breast carcinoma cells. Lab Invest 83: 435–448 12649344 10.1097/01.lab.0000059927.97515.fd

[bib2] Angelucci A, Gravina GL, Rucci N, Millimaggi D, Festuccia C, Muzi P, Teti A, Vicentini C, Bologna M (2006) Suppression of EGF-R signaling reduces the incidence of prostate cancer metastasis in nude mice. Endocr Relat Cancer 13: 197–210 16601288 10.1677/erc.1.01100

[bib3] Bates RC, Mercurio AM (2005) The epithelial–mesenchymal transition (EMT) and colorectal cancer progression. Cancer Biol Ther 4: 365–370 15846061 10.4161/cbt.4.4.1655

[bib4] Brabletz T, Jung A, Reu S, Porzner M, Hlubek F, Kunz-Schughart LA, Knuechel R, Kirchner T (2001) Variable beta-catenin expression in colorectal cancers indicates tumor progression driven by the tumor environment. Proc Natl Acad Sci USA 98: 10356–10361 11526241 10.1073/pnas.171610498 PMC56965

[bib5] Bryden AA, Hoyland JA, Freemont AJ, Clarke NW, Schembri-Wismayer D, George NJ (2002) E-cadherin and beta catenin are down-regulated in prostatic bone mestatases. BJU Intl 89: 400–403 10.1046/j.1464-4096.2001.01712.x 11872032

[bib6] Chaffer CL, Brennan JP, Slavin JL, Blick T, Thompson EW, Williams ED (2006) Mesenchymal-to-epithelial transition facilitates bladder cancer metastasis: role of fibroblast growth factor receptor-2. Cancer Res 66: 11271–11278 17145872 10.1158/0008-5472.CAN-06-2044

[bib7] Condeelis J, Segall JE (2003) Intravital imaging of cell movement in tumours. Nat Rev Cancer 3: 921–930 14737122 10.1038/nrc1231

[bib8] Davies G, Jiang G, Mason MD (2000) Cell–cell adhesion molecules and signaling intermediates and their role in the invasive potential of prostate cancer cells. J Urol 163: 985–992 10688036

[bib9] Ewing J (1922) Tumors of prostate. In Neoplastic Diseases pp 784–785. Philadelphia and London: WB Saunders Company

[bib10] Gingrich JR, Barrios RJ, Morton RA, Boyce BF, DeMayo FJ, Finegold MJ, Angelopoulou R, Rosen JM, Greenburg NM (1996) Metastatic prostate cancer in a transgenic mouse. Cancer Res 56: 4096–4102 8797572

[bib11] Graff JR, Herman JG, Lapidus RG, Chopra H, Xu R, Jarrard DF, Isaacs WB, Pitha PM, Davidson NE, Baylin SB (1995) E-cadherin expression is silenced by DNA hypermethylation in human breast and prostate carcinomas. Cancer Res 55: 5195–5199 7585573

[bib12] Hurtubise A, Momparler RL (2004) Evaluation of antineoplastic action of 5-aza-2′-deoxycytidine (Dacogen) and docetaxel (Taxotere) on human breast, lung and prostate carcinoma cell lines. Anticancer Drugs 15: 161–167 15075673 10.1097/00001813-200402000-00010

[bib13] Imai T, Horiuchi A, Shiozawa T, Osada R, Kikuchi N, Ohira S, Oka K, Konishi I (2004) Elevated expression of E-cadherin and alpha-, beta-, and gamma-catenins in metastatic lesions compared with primary epithelial ovarian carcinomas. Human Pathol 33: 1469–1476 10.1016/j.humpath.2004.09.014 15619205

[bib14] Jawhari AU, Farthing MJ, Pignatelli M (1999) The E-cadherin/epidermal growth factor receptor interaction: a hypothesis of reciprocal and reversible control of intercellular adhesion and cell proliferation. J Pathol 187: 155–157 10365089 10.1002/(SICI)1096-9896(199901)187:2<155::AID-PATH193>3.0.CO;2-E

[bib15] Kallakury BV, Sheehan CE, Winn-Deen E, Oliver J, Fisher HA, Kaufman RP, Ross JS (2001) Decreased expression of catenins (alpha and beta), p120 CTN, and E-cadherin cell adhesion proteins and E-cadherin gene promoter methylation in prostatic adenocarcinomas. Cancer 92: 2786–2795 11753952 10.1002/1097-0142(20011201)92:11<2786::aid-cncr10128>3.0.co;2-i

[bib16] Kowalski PJ, Rubin MA, Kleer CG (2003) E-cadherin expression in primary carcinomas of the breast and its distant metastases. Breast Cancer Res 5: R217–R222 14580257 10.1186/bcr651 PMC314411

[bib17] Lind GE, Thorstensen L, Lovig T, Meling GI, Hamelin R, Rognum TO, Esteller M, Lothe RA (2004) A CpG island hypermethylation profile of primary colorectal carcinomas and colon cancer cell lines. Mol Cancer 3: 28 15476557 10.1186/1476-4598-3-28 PMC526388

[bib18] Lowy AM, Knight J, Groden J (2002) Restoration of E-cadherin/beta-catenin expression in pancreatic cancer cells inhibits growth by induction of apoptosis. Surgery 132: 141–148 12219004 10.1067/msy.2002.125168

[bib19] McClay DR, Wessel GM, Marchase RB (1981) Intercellular recognition: quantitation of initial binding events. Proc Natl Acad Sci USA 78: 4975–4979 6946443 10.1073/pnas.78.8.4975 PMC320314

[bib20] Rubin MA, Mucci NR, Figurski J, Fecko A, Pienta KJ, Day ML (2001) E-cadherin expression in prostate cancer: a broad survey using high-density tissue microarray technology. Hum Pathol 32: 690–697 11486167 10.1053/hupa.2001.25902

[bib21] Seglen PO (1976) Preparation of isolated rat liver cells. Methods Cell Biol 13: 29–83 177845 10.1016/s0091-679x(08)61797-5

[bib22] Shah RB, Mehra R, Chinnaiyan AM, Shen R, Ghosh D, Zhou M, Macvicar GR, Varambally S, Harwood J, Bismar TA, Kim R, Rubin MA, Pienta KJ (2004) Androgen-independent prostate cancer is a heterogeneous group of diseases: lessons from a rapid autopsy program. Cancer Res 64: 9209–9216 15604294 10.1158/0008-5472.CAN-04-2442

[bib23] Thiery JP (2002) Epithelial–mesenchymal transitions in tumour progression. Nat Rev Cancer 2: 442–454 12189386 10.1038/nrc822

[bib24] Wells A (2000) Tumor invasion: role of growth factor-induced cell motility. Adv Cancer Res 78: 31–101 10547668 10.1016/s0065-230x(08)61023-4

[bib25] Welsh JB, Gill GN, Rosenfeld MG, Wells A (1991) A negative feedback loop attenuates EGF-induced morphological changes. J Cell Biol 114: 533–543 1860884 10.1083/jcb.114.3.533 PMC2289101

[bib26] Wheeler JM (2005) Epigenetics, mismatch repair genes and colorectal cancer. Ann R Coll Surg Engl 87: 15–20 15720901 10.1308/1478708051423 PMC1963859

[bib27] Wong AS, Gumbiner BM (2003) Adhesion-independent mechanism for suppression of tumor cell invasion by E-cadherin. J Cell Biol 161: 1191–1203 12810698 10.1083/jcb.200212033 PMC2173007

[bib28] Yates C, Shepard C, Papworth G, Dash A, Beer-Stolz D, Griffith L, Wells A (2007) Novel three-dimensional organotypic liver bioreactor to directly visualize early events in metastatic progression. Adv Cancer Res 96 (in press) 10.1016/S0065-230X(06)97010-9 17419948

[bib29] Yates C, Wells A, Turner T (2005) Luteinising hormone-releasing hormone analogue reverses the cell adhesion profile of EGFR overexpressing DU-145 human prostate carcinoma subline. Br J Cancer 92: 366–375 15655536 10.1038/sj.bjc.6602350 PMC2361841

